# Neck Circumference May Be a Better Alternative to Standard Anthropometric Measures

**DOI:** 10.1155/2016/6058916

**Published:** 2016-02-11

**Authors:** Kaumudi Joshipura, Francisco Muñoz-Torres, José Vergara, Cristina Palacios, Cynthia M. Pérez

**Affiliations:** ^1^Center for Clinical Research and Health Promotion, University of Puerto Rico, Medical Sciences Campus, San Juan, PR 00936-5067, USA; ^2^Department of Epidemiology, Harvard T.H. Chan School of Public Health, Boston, MA 02115, USA; ^3^Graduate School of Public Health, University of Puerto Rico, Medical Sciences Campus, San Juan, PR 00936-5067, USA

## Abstract

This paper evaluates neck circumference as a metabolic risk marker. Overweight/obese, nondiabetic Hispanics, 40–65 years old, who are free of major cardiovascular diseases, were recruited for the San Juan Overweight Adults Longitudinal Study (SOALS). Baseline exams were completed by 1,206 participants. Partial correlation coefficients (*r*) and logistic models adjusted for age, gender, smoking status, and physical activity were computed. Neck circumference was significantly correlated with waist circumference (*r* = 0.64), BMI (*r* = 0.66), and body fat % (*r* = 0.45). Neck circumference, highest (compared to lowest) tertile, had higher association with prediabetes: multivariable OR = 2.30 (95% CI: 1.71–3.06) compared to waist circumference OR = 1.97 (95% CI: 1.48–2.66) and other anthropometric measures. Neck circumference showed higher associations with HOMA, low HDL-C, and triglycerides, multivariable OR = 8.42 (95% CI: 5.43–13.06), 2.41 (95% CI: 1.80–3.21), and 1.52 (95% CI: 1.14–2.03), but weaker associations with hs-CRP and hypertension, OR = 3.61 (95% CI: 2.66–4.90) and OR = 2.58 (95% CI: 1.90–3.49), compared to waist circumference. AIC for model fit was generally similar for neck or waist circumference. Neck circumference showed similar or better associations with metabolic factors and is more practicable than waist circumference. Hence, neck circumference may be a better alternative to waist circumference.

## 1. Introduction

Obesity is rising to pandemic proportions [1] and is an important risk factor for cardiometabolic diseases, including diabetes, hypertension, dyslipidemia, and coronary heart disease (CHD) [2–4]. Neck circumference may likely be a very convenient and valid alternative measure of obesity and may even be a better marker of metabolic risk compared to standard measures such as body mass index (BMI) and waist circumference (WC). Overweight and obesity are defined by BMI, depicting weight higher than what is generally considered healthy for a given height [5], although the location of fat may modify the health implications of BMI, and central obesity is generally considered to be a stronger risk factor for cardiometabolic risk than overall obesity [6]. An upper body distribution of fat, especially with increased visceral adipose tissue, is considered predictive of cardiometabolic conditions [7–9]. Several anthropometric measures are used to assess overall obesity and also to assess specific aspects such as central or abdominal obesity, visceral fat, or subcutaneous fat. Computed tomography and MRI are the gold standard methods for measuring visceral fat, and dual-energy X-ray absorptiometry is considered a very reliable alternative [10]. However, these are expensive and not feasible for large epidemiological studies [11]. Anthropometric measures such as weight and height for calculating BMI and waist circumference are relatively low-burden standard and valid surrogate measures for abdominal adiposity.

Overweight and obesity may be associated with fat deposition in the neck [12], resulting in higher neck circumference. Neck circumference is a simple, convenient but less used anthropometric measure, which is correlated with waist circumference and BMI, and has been associated with components of metabolic syndrome in cross-sectional [12–17] and cohort studies in different populations [14, 18]. The association between neck fat and metabolic syndrome and its components may be attributed to an excess release of free fatty acids into plasma from the upper body subcutaneous fat [9]. High levels of plasma free fatty acids, in turn, have been associated with markers of oxidative stress and insulin resistance [19], which in turn impact glycemia. It has been suggested that fat in the neck may be more similar to visceral fat, which is more strongly related with cardiometabolic risks compared to subcutaneous fat [9]. An increase in neck circumference has even been found to be an independent predictor of nonalcoholic fatty liver disease [20–22]. Although studies have shown associations between neck circumference and components of metabolic syndrome, more studies directly comparing neck circumference with other anthropometric measures are needed, as neck circumference is not included in standard guidelines and practices and is not generally included in research studies or clinical assessments [23, 24], even in situations when waist circumference may not be feasible or meaningful. Neck circumference is rarely evaluated in clinical practice or research, although it is a more practical and likely better measure, which may be especially useful in special populations such as morbidly obese people, patients in bed rest, and pregnant women. Hence more studies are needed in different populations to elucidate the utility of this measurement for assessing obesity and predicting cardiometabolic risk factors when the traditional anthropometric measurements are not practicable or valid. Therefore, the aim of this paper is to compare the relative utility of neck circumference as a metabolic risk marker among a high risk group of overweight/obese Hispanic adults by comparing associations of neck circumference and metabolic factors including metabolic syndrome components, against similar comparisons using waist circumference and other standard measures.

## 2. Methods and Procedures

### 2.1. Study Sample

**Figure 1:**
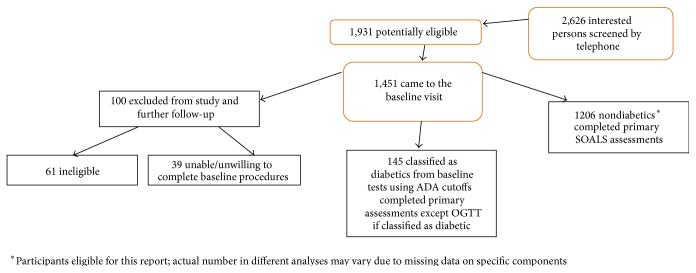
Description of SOALS screening and exclusions.

The present study utilizes baseline data from the San Juan Overweight Adults Longitudinal Study (SOALS). The study was approved by the University of Puerto Rico Institutional Review Board. Participants who were free of previously diagnosed diabetes, between 40 and 65 years, and overweight or obese (BMI ≥ 25.0 kg/m^2^) were recruited primarily from San Juan municipality area. Screening and exclusion criteria for participants are described in Figure 1. People were excluded if they had previously diagnosed diabetes, had braces or less than four teeth (since one of the primary goals of SOALS was relating periodontitis and glucose abnormalities), were pregnant, had some systemic conditions (such as physician-diagnosed hypoglycemia, congenital heart murmurs, heart valve disease, congenital heart disease, endocarditis, rheumatic fever, and hemophilia or bleeding disorders), or were unable to complete study procedures. Most exclusion criteria relate to health conditions that could potentially increase the risk of systemic complications that could develop during a periodontal examination. Participants were further excluded if they had fasting plasma glucose ≥ 126 mg/dL, two-hour oral glucose tolerance test (OGTT) ≥ 200 mg/dL, or glycosylated hemoglobin (HbA1c) ≥ 6.5% at the baseline exam [25]. The 1,206 eligible participants who completed the baseline exam were included in the analyses.

### 2.2. Anthropometric Measures

Anthropometric measurements were taken in duplicate according to the NHANES III procedures. In cases where the first two measures differed by 0.5 cm, a third measure was recorded, and the average of all measures recorded was computed. Both body weight (0.2 kg weight graduation) and body fat percent (0.1% body fat graduation) were measured by a bioelectrical impedance analysis (BIA) technology using Tanita scale (Tanita Body Composition Analyzer-TBF-310A), and height was measured in meters using a portable stadiometer (Seca Corporation, Hanover, MD) to calculate BMI. Circumferences were measured with a Gulick tape. Waist circumference was measured at the umbilicus and recorded to the nearest 0.1 cm. Neck circumference was measured below the laryngeal prominence and perpendicular to the long axis of the neck, and the minimal circumference was recorded to the nearest 0.1 cm [26]. The average Pearson correlation coefficient between repeats for all measures was greater than 0.99 showing excellent reproducibility.

### 2.3. Metabolic Measures

We used standard cutoffs from the literature as described below for classifying metabolic syndrome and its components [27]. Participants were classified as having elevated blood pressure if they had systolic blood pressure ≥ 130 mm Hg or diastolic blood pressure ≥ 85 mm Hg or reported antihypertensive drug treatment. Elevated triglycerides were defined as levels ≥ 150 mg/dL or a history of drug treatment for elevated triglycerides. Low HDL-C was defined as levels < 40 mg/dL in men and levels < 50 mg/dL in women or history of drug treatment for reduced HDL-C. Elevated fasting glucose was defined as levels ≥ 100 mg/dL or a history of drug treatment for elevated glucose.

Individuals with adiposity or metabolic syndrome commonly manifest insulin resistance, prediabetes, and a proinflammatory state [27]; hence these were also evaluated. High sensitivity C-reactive protein (hs-CRP) levels were considered high if they exceeded 3 mg/L. Glucose and insulin levels were evaluated at fasting and after administration of a 75 g glucose load at 30, 60, and 120 minutes. Glucose was measured using an enzymatic colorimetric assay. Plasma insulin concentrations were analyzed using an immunochemiluminometric assay. Insulin resistance was estimated using HOMA-IR [fasting glucose × fasting insulin/405]. (HbA1C) was measured with an assay based on a latex immunoagglutination inhibition method (DCA 2000+ Analyzer, Siemens Healthcare Diagnostics, NY, US). Prediabetes was defined using standard cutoffs as fasting plasma glucose within 100–125 mg/dL, 2 hr OGTT 140–199 mg/dL, or HbA1C of 5.7–6.4% [25].

### 2.4. Other Measures

A questionnaire was administered by trained interviewers and included sociodemographic characteristics, lifestyle factors including diet, physical activity, and sleep duration and disorders, and medical and family history including medication use. Sleep disordered breathing (SDB) was assessed by reported physician diagnosis of sleep apnea, insomnia, and restless legs syndrome. Physical activity was defined as at least 150 minutes of moderate-intensity aerobic physical activity per week or at least 75 minutes of vigorous-intensity aerobic physical activity per week or an equivalent combination of moderate- and vigorous-intensity activity (WHO recommended levels of physical activity for adults aged 18–64 years).

### 2.5. Statistical Analyses

Baseline characteristics of study participants were compared by high and normal neck circumference categories using Student's *t*-test, Mann-Whitney-Wilcoxon test, or chi square test. Since there were no standard thresholds, cutoffs were based on the median by gender: ≥35.8 cm for women and ≥41.3 cm for men. Potential confounders for these associations were selected a priori based on the literature. Partial Pearson's correlations, adjusted for age, gender, smoking status, and physical activity, were calculated between anthropometric measures (neck circumference, waist circumference, BMI, and body fat percent) and between anthropometric measures and metabolic factors including components of metabolic syndrome. Partial correlations were selected as they are easier to conceptualize and do not distinguish between exposure and outcome.

Logistic regression models were fit separately to evaluate the association of tertiles (calculated separately by gender and then combined) for the different anthropometric measurements with binary outcomes including prediabetes, HOMA-IR (defined as the upper quartile of the HOMA-IR distribution), elevated blood pressure, elevated triglycerides, low HDL-C, and elevated hs-CRP. Although the scale and distribution differ across different anthropometric measures, tertiles would uniformly compare the highest third versus the lowest third. All models were adjusted for age, gender, smoking status, and physical activity. The Akaike Information Criterion (AIC) was used to determine which of the candidate models best approximated the data (lower values indicating better fit).

## 3. Results

Individuals with high and normal neck circumference were similar in age and gender (Table 1). The mean neck circumference was 42.0 ± 4.8 cm for men and 36.1 ± 2.9 cm for women (not shown in the table). Among those with high neck circumference, 16% were current smokers compared to 22% in those with normal neck circumference. As expected, the group with high neck circumference had higher BMI, waist circumference, and body fat percent. Measures of glucose tolerance, HOMA-IR, triglycerides, and hs-CRP were also higher among individuals with high neck circumference compared to normal neck circumference subjects. The percent of individuals with prediabetes, hypertension, and metabolic syndrome were higher among the group with high compared to normal neck circumference. Individuals in the normal neck circumference group had higher physical activity and HDL-C than those in the higher neck circumference group.

**Figure 2:**
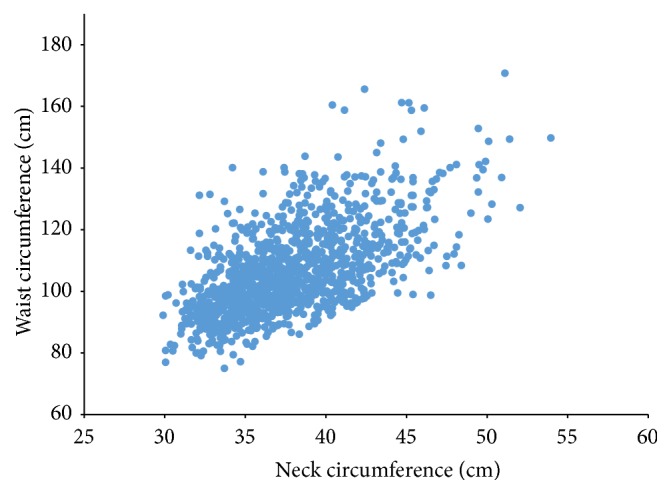
Plot relating waist and neck circumferences.

Table 2 shows Pearson's partial correlation coefficients between anthropometric measures, adjusted for age, gender, smoking status, and physical activity. BMI showed the largest correlation with waist circumference compared to its correlations with other anthropometric measures (*r* = 0.87, *p* < 0.001). Neck circumference was significantly (*p* < 0.001) correlated with traditional assessments of body composition such as BMI (*r* = 0.66), waist circumference (*r* = 0.64), and body fat percent (*r* = 0.45).  Figure 2 shows a linear relationship between neck and waist circumference, with the spread increasing with increasing circumferences.  Table 3 shows partial correlation coefficients between anthropometric measures and metabolic factors. hs-CRP showed lowest correlations with neck circumference (*r* = 0.30) compared to waist (*r* = 0.40) and BMI (*r* = 0.46), and body fat percent showed larger correlation with fasting glucose (*r* = 0.14) compared to neck (*r* = 0.10). All other metabolic factors showed significant correlations with most anthropometric measures with the highest correlations with neck circumference: 1 hr OGTT (*r* = 0.18), 2 hr OGTT (*r* = 0.10), HbA1c (*r* = 0.28), HOMA-IR (*r* = 0.45), and systolic (*r* = 0.18) and diastolic (*r* = 0.23) blood pressure, and HDL-C (*r* = −0.23); triglycerides were significantly associated only with neck with a small correlation of 0.12. Neck circumference was not adjusted for height since adjustment did not improve the correlations with other anthropometric measures or with glucose abnormalities.

Multivariable logistic regression analysis showed that individuals classified in the middle and upper tertiles (cutoffs described in Table 4) of neck circumference exhibited higher odds of prediabetes (OR = 1.34, 95% CI: 1.01–1.79; OR = 2.30, 95% CI: 1.71–3.06, resp.) compared to those in the lowest tertile after adjusting for age, gender, smoking status, and physical activity (Table 5). Positive associations of prediabetes with middle and upper tertiles of waist circumference (OR = 1.22, 95% CI: 0.91–1.61; OR = 1.97, 95% CI: 1.48–2.66), BMI (OR = 1.41, 95% CI: 1.06–1.87; OR = 2.00, 95% CI: 1.49–2.68), and body fat percent (OR = 1.27, 95% CI: 0.95–1.67; OR = 1.82, 95% CI: 1.36–2.46, resp.) were generally weaker, and models for other anthropometric measures showed higher AIC (1578–1585) indicating worse model fit compared to neck (AIC = 1574). Only 52 participants reported SDB and the associations between neck circumference and insulin resistance (or prediabetes) did not change after adjusting for SDB. Since our previous work in this population showed that 1 hr postload glucose was a better metabolic marker than 2 hr glucose [28], we evaluated an additional measure of prediabetes. In multivariable analyses comparing the upper tertile of neck circumference, waist circumference, BMI, and body fat percent with the lowest tertile, the odds of prediabetes increased after adding the 1 hr glucose > 155 mg/dL to the prediabetes definition: OR = 2.63, 2.19, 2.40, and 2.10, respectively (data not shown in tables), compared to OR = 2.30, 1.97, 2.00, and 1.82, respectively, with prediabetes defined by only fasting glucose, 2 hr glucose, or HbA1c.

The upper tertile of neck circumference exhibited higher odds of being in the highest quartile (versus lower 3 quartiles) of HOMA-IR (OR = 8.42, 95% CI: 5.43–13.06) compared to waist (OR = 7.99, 95% CI: 5.08–12.57). Neck circumference also showed higher inverse association with low HDL-C (OR = 2.41 comparing highest and lowest tertile, 95% CI: 1.80–3.21) compared to waist circumference (OR = 2.13, 95% CI: 1.59–2.84) and other anthropometric measures. Compared to other anthropometric measures, upper tertiles of BMI had higher association with hs-CRP (OR = 6.76, 95% CI: 4.87–9.39).

## 4. Discussion

The present study shows that neck circumference was significantly associated with measures of overall and central adiposity; the magnitude of the associations is modest ranging from 0.45 to 0.66. The correlations relating both neck and other anthropometric measures with metabolic factors are below 0.47 suggesting modest to weak correlations that are similar or higher for neck circumference (except with hs-CRP and fasting glucose). Importantly, compared to traditional anthropometric measures such as BMI, waist circumference, and body fat percent, neck circumference showed higher positive associations with prediabetes and higher inverse association with HDL-C, independent of major confounders. As with waist circumference, neck circumference seems to be a good measure without adjusting for height, as the associations were similar when we considered factoring variations in height by using neck to height ratio.

Waist circumference is a widely used anthropometric measure reflecting central obesity, a major risk factor for cardiometabolic conditions. However, this measurement requires training for it to be reliable. Also, the cutoff points for high WC were derived from regression analysis identifying WC values associated with obesity based on BMI, so its incremental value beyond BMI may be somewhat limited [2]. On the other hand, neck circumference is a simpler and more practical anthropometric parameter, not impeded by clothing or last meal. Our data also shows that neck circumference has a smaller correlation with BMI, compared to waist circumference, implying that the incremental value of adding neck circumference would be higher, as neck circumference would be more independent of BMI compared to waist circumference. Neck circumference should be included in guidelines and recommended for assessing obesity, especially in situations when the traditional anthropometric measures are not available, convenient, feasible, or meaningful. Our study adds to the evidence that it is well correlated with other anthropometric measures and may be a good marker for glucose homeostasis parameters [12, 13, 29] and metabolic conditions [9, 12–14, 30], including blood pressure, fasting plasma glucose levels, and insulin resistance, and may be a good surrogate for visceral adiposity. Neck circumference was associated with hs-CRP and also correlated with triglycerides and inversely related with HDL-C even after adjusting for BMI or waist circumference [31, 32].

It has been suggested that sleep disordered breathing might mediate the association between neck circumference and cardiometabolic risk factors [9, 33] independent of obesity [34]. The associations between neck circumference and insulin resistance (or prediabetes) did not change after adjusting for SDB in our study, suggesting that SDB is not mediating the associations, possibly because of the low number of participants who self-reported SDB. It has been hypothesized that fat in the neck, more similar to visceral fat, produces and releases substances that cause metabolic abnormalities [35]. More recently it has been described as an ectopic fat depot functioning as a reserve for immediate energy source [36] in a location not typically associated with adipose tissue storage. This causes increased delivery of free fatty acids to the liver, causing oxidative stress [37] and ultimately leading to an increased cardiovascular and metabolic risk [38, 39]. Another likely pathway is that subcutaneous fat in the upper body, particularly in obese individuals, is responsible for a much bigger proportion of release of free fatty acids than visceral adipose tissue [40], and high levels of plasma free fatty acids could result in insulin resistance [19].

The cross-sectional data limits us from evaluating the role of neck circumference in predicting incidence or progression of metabolic conditions. However, given the significant and consistent associations in our study and other populations, neck circumference shows promise as an alternative marker for the risk associated with central or visceral adiposity. Measurement of neck circumference has been proposed as a useful tool for clinical screening of persons with a high risk of insulin resistance [41].

Further prospective research is needed to evaluate whether neck circumference is an important risk factor for the development of cardiometabolic conditions. Additional work is needed to understand whether neck circumference can substitute or add to more traditional anthropometric measures such as waist circumference and to develop composite anthropometric measures incorporating neck circumference. The utility of neck circumference may be higher among populations where waist circumference is hard to measure or not interpretable as a measure of central adiposity because of culture, time of the day, clothing, last meal, empty bladder, pregnancy, and various health conditions, all of which are unlikely to impact neck circumference.

## 5. Conclusion

This cross-sectional study shows that neck circumference has higher associations with prediabetes and lower associations for hs-CRP compared to traditional anthropometric measures, and the associations with other metabolic factors are generally similar to waist circumference. Neck circumference may be an important marker of central adiposity and perhaps of visceral adiposity and an important risk indicator of metabolic conditions. Neck circumference may be an important measure to consider for routine assessment in primary care clinics and other health care settings as well as for research studies when the use of expensive and sophisticated machines is neither easy nor justifiable. It may be especially useful among populations such as pregnant women where traditional measures may be challenging or not meaningful.

## Figures and Tables

**Table 1:** Baseline characteristics according to neck circumference categories.

	Neck circumference		
	Normal	High	*p* value^*∗*^
*N* ^†^	601	605	—
Age (years)	50.0	50.0	**0.10**
Male gender (%)	27.3	27.3	**1.00**
Current smoker (%)	22.0	16.4	**0.007**
Physical activity (%)	57.2	49.9	**0.01**
Body mass index (kg/m^2^)	29.2	35.3	**<0.001**
Neck circumference (cm)	34.7	38.8	**<0.001**
Waist circumference (cm)	98.4	111.3	**<0.001**
Body fat percent	37.7	42.5	**<0.001**
HOMA-IR index	1.6	2.7	**<0.001**
Fasting glucose (mg/dL)	91.0	93.0	**0.01**
1 hr OGTT (mg/dL)	146.0	160.0	**<0.001**
2 hr OGTT (mg/dL)	109.0	115.0	**<0.001**
HbA1c	5.6	5.8	**<0.001**
Prediabetes (%)	50.8	64.1	**<0.001**
Systolic blood pressure (mm Hg)	123.3	128.7	**<0.001**
Diastolic blood pressure (mm Hg)	78.7	82.0	**<0.001**
Hypertension (%)	49.9	62.6	**<0.001**
HDL-C (mg/dL)	49.0	44.0	**<0.001**
Triglycerides (mg/dL)	120.0	136.0	**<0.001**
hs-CRP (mg/L)	2.8	5.5	**<0.001**
Metabolic syndrome (%)	38.6	60.0	**<0.001**

^*∗*^ *p* values comparing high and low neck circumference groups by Student's *t*-test, Mann-Whitney-Wilcoxon test, or chi square test. Medians were computed where the distributions were not normal.

^†^Sample sizes varied across measures where there were missing values.

**Table 2:** Pearson's partial correlations between anthropometric measurements^*∗*^.

	Neck circumference	Waist circumference	BMI
Waist circumference	0.64^†^	—	—
BMI	0.66^†^	0.87^†^	—
Body fat %	0.45^†^	0.62^†^	0.65^†^

^*∗*^Adjusted for age, gender, smoking status, and physical activity.

^†^ *p* < 0.001.

**Table 3:** Pearson's partial correlations of anthropometric measurements with metabolic factors^*∗*^.

	Neck circumference	Waist circumference	BMI	Body fat percent
HOMA-IR	0.45^‡^	0.41^‡^	0.41^‡^	0.33^‡^
Fasting glucose	0.10^†^	0.11^‡^	0.09^†^	0.14^‡^
1 hr OGTT	0.18^‡^	0.12^‡^	0.11^‡^	0.16^‡^
2 hr OGTT	0.10^†^	0.04	0.06^†^	0.09^†^
HbA1c	0.28^‡^	0.23^‡^	0.23^‡^	0.18^‡^
Systolic blood pressure	0.18^‡^	0.16^‡^	0.16^‡^	0.08^†^
Diastolic blood pressure	0.23^‡^	0.19^‡^	0.18^‡^	0.13^‡^
HDL-C	−0.23^‡^	−0.18^‡^	−0.14^†^	−0.01
Triglycerides	0.12^†^	0.02	−0.02	0.00
hs-CRP	0.30^‡^	0.40^‡^	0.46^‡^	0.33^‡^

^*∗*^Adjusted for age, gender, smoking status, and physical activity.

^†^ *p* < 0.05, ^‡^ *p* < 0.001.

**Table 4:** Ranges for tertiles of anthropometric measures.

	Tertile	Male	Female
Neck circumference	1st	32.2, 40.1	29.9, 34.6
	2nd	40.2, 42.8	34.7, 37.0
	3rd	42.9, 54.0	37.1, 48.4

Waist circumference	1st	86.1, 102.3	75.0, 97.4
	2nd	102.4, 114.3	97.5, 108.2
	3rd	114.4, 170.8	108.3, 199.7

BMI	1st	25.3, 29.1	25.0, 29.6
	2nd	29.2, 34.1	29.7, 34.7
	3rd	34.2, 67.8	34.8, 65.9

Body fat percent	1st	12.6, 26.7	13.6, 39.3
	2nd	26.8, 34.0	39.4, 44.6
	3rd	34.1, 63.7	44.7, 63.6

**Table 5:** Logistic regression for associations between anthropometric measures and metabolic measures including components of metabolic syndrome^*∗∗*^.

Binary metabolic outcomes	Anthropometric measure tertile	Neck circumference		Waist circumference		BMI		Body fat percent	
	Tertile	OR	95% CI	OR	95% CI	OR	95% CI	OR	95% CI
Hypertension	1	1.00	—	1.00	—	1.00	—	1.00	—
	2	1.35^*∗*^	1.01–1.81	1.68^*∗*^	1.25–2.25	1.61^*∗*^	1.20–2.16	1.53^*∗*^	1.14–2.05
	3	2.58^*∗*^	1.90–3.49	2.76^*∗*^	2.03–3.75	2.78^*∗*^	2.05–3.79	2.55^*∗*^	1.88–3.46

	AIC^*λ*^	1511.05		1507.60		1507.24		1503.10	

Low HDL-C	1	1.00	—	1.00	—	1.00	—	1.00	—
	2	1.50^*∗*^	1.13–1.99	1.96^*∗*^	1.47–2.61	1.48^*∗*^	1.12–1.97	0.91	0.69–1.21
	3	2.41^*∗*^	1.80–3.21	2.13^*∗*^	1.59–2.84	1.87^*∗*^	1.41–2.50	1.07	0.80–1.42

	AIC	1622.27		1626.60		1639.99		1646.83	

Triglycerides ≥ 150	1	1.00	—	1.00	—	1.00	—	1.00	—
	2	1.15	0.86–1.55	1.16	0.87–1.55	1.26	0.94–1.69	1.28	0.96–1.71
	3	1.52^*∗*^	1.14–2.03	1.23	0.92–1.65	1.27	0.94–1.70	1.16	0.87–1.56

	AIC	1586.28		1592.46		1591.26		1581.61	

Glucose ≥ 100	1	1.00	—	1.00	—	1.00	—	1.00	—
	2	1.08	0.76–1.53	1.21	0.85–1.72	1.13	0.79–1.60	1.52^*∗*^	1.05–2.20
	3	1.18	0.84–1.67	1.45^*∗*^	1.02–2.05	1.26	0.89–1.79	1.97^*∗*^	1.37–2.83

	AIC	1224.50		1221.13		1223.71		1198.65	

hs-CRP > 3	1	1.00	—	1.00	—	1.00	—	1.00	—
	2	1.78^*∗*^	1.34–2.38	1.83^*∗*^	1.38–2.44	2.42^*∗*^	1.81–3.24	1.43^*∗*^	1.08–1.91
	3	3.61^*∗*^	2.66–4.90	5.20^*∗*^	3.78–7.16	6.76^*∗*^	4.87–9.39	4.29^*∗*^	3.13–5.87

	AIC	1528.21		1486.83		1452.85		1494.83	

HOMA-IR^†^	1	1.00	—	1.00	–	1.00	—	1.00	—
	2	1.97^*∗*^	1.21–3.17	2.82^*∗*^	1.74–4.57	4.46^*∗*^	2.63–7.58	2.09^*∗*^	1.35–3.23
	3	8.42^*∗*^	5.43–13.06	7.99^*∗*^	5.08–12.57	10.95^*∗*^	6.58–18.24	4.86^*∗*^	3.24–7.33

	AIC	1035.89		1062.43		1047.45		1096.29	

Prediabetes^‡^	1	1.00	—	1.00	—	1.00	—	1.00	—
	2	1.34^*∗*^	1.01–1.79	1.22	0.91–1.61	1.41^*∗*^	1.06–1.87	1.27	0.95–1.67
	3	2.30^*∗*^	1.71–3.06	1.97^*∗*^	1.48–2.66	2.00^*∗*^	1.49–2.68	1.82^*∗*^	1.36–2.46

	AIC	1574.19		1584.42		1584.66		1578.04	

^*∗∗*^Adjusted for age, gender, smoking status, and physical activity.

^*∗*^ *p* < 0.05.

^*λ*^Model selection criteria: lower AIC implies better model fitness.

^†^Upper quartile versus lower three quartiles for HOMA-IR cutoff.

^‡^Prediabetes versus normal glycemia defined by ADA plasma fasting glucose, 2 hr OGTT, and HbA1c cutoffs.
